# Clinical Potential of DNA Methylation in Gastric Cancer: A Meta-Analysis

**DOI:** 10.1371/journal.pone.0036275

**Published:** 2012-04-27

**Authors:** Nur Sabrina Sapari, Marie Loh, Aparna Vaithilingam, Richie Soong

**Affiliations:** 1 Cancer Science Institute of Singapore, National University of Singapore, Singapore, Singapore; 2 School of Surgery, University of Western Australia, Crawley, Australia; 3 Department of Pathology, National University of Singapore, Singapore, Singapore; Ospedale Pediatrico Bambino Gesu', Italy

## Abstract

**Background:**

Accumulating evidence indicates aberrant DNA methylation is involved in gastric tumourigenesis, suggesting it may be a useful clinical biomarker for the disease. The aim of this study was to consolidate and summarize published data on the potential of methylation in gastric cancer (GC) risk prediction, prognostication and prediction of treatment response.

**Methods:**

Relevant studies were identified from PubMed using a systematic search approach. Results were summarized by meta-analysis. Mantel-Haenszel odds ratios were computed for each methylation event assuming the random-effects model.

**Results:**

A review of 589 retrieved publications identified 415 relevant articles, including 143 case-control studies on gene methylation of 142 individual genes in GC clinical samples. A total of 77 genes were significantly differentially methylated between tumour and normal gastric tissue from GC subjects, of which data on 62 was derived from single studies. Methylation of 15, 4 and 7 genes in normal gastric tissue, plasma and serum respectively was significantly different in frequency between GC and non-cancer subjects. A prognostic significance was reported for 18 genes and predictive significance was reported for *p16* methylation, although many inconsistent findings were also observed. No bias due to assay, use of fixed tissue or CpG sites analysed was detected, however a slight bias towards publication of positive findings was observed.

**Conclusions:**

DNA methylation is a promising biomarker for GC risk prediction and prognostication. Further focused validation of candidate methylation markers in independent cohorts is required to develop its clinical potential.

## Introduction

Gastric cancer (GC) remains a major clinical challenge worldwide due to its high prevalence, poor prognosis and limited treatment options [1]. Although the incidence of GC has declined over the years, it continues to be the second leading cause of cancer death and the fourth most common malignancy worldwide. Less than 25% of GC cases are diagnosed at an early stage, and the 5-year survival rate is only 24% in the US and Europe [2]. However, the survival rate from GC improves to over 60% if detected at an early stage [2], emphasizing the importance of early detection in this cancer type.

DNA methylation is an epigenetic mechanism of transcriptional regulation, with an involvement in cancer attributed to the inappropriate silencing of tumour suppressor genes, or loss of oncogene repression [3]. Since the first article by Fang *et al.* in 1996 describing DNA hypomethylation of c-myc and c-Ha-ras in GC [4], more than 550 studies have been published on the involvement of aberrant DNA methylation in the development of GC. As a result, the presence and functional consequences of aberrant DNA methylation of more than 100 genes in GC has been reported [5]–[17]. Evidence on links between aberrant DNA methylation to *H. pylori* infection [1], [18]–[22] and its involvement in precancerous gastric epithelial lesions and GC progression [18], [19], [21], [23]–[25] are also being increasingly documented. Taken together, these results have indicated aberrant DNA methylation has a significant role in gastric cancer development and progression.

The pattern of tumour DNA methylation can be useful for cancer risk screening, prognostication and treatment prediction [3], [26]–[30]. Compared to somatic mutation, DNA methylation has a higher number of aberrant alterations per cancer cell [31]. Moreover, aberrant DNA methylation occurs early in the tumourigenesis of many cancer types [28], making it particularly useful for risk prediction. The technical attraction of DNA methylation is that it is chemically stable and can be detected with a very high sensitivity of up to 1∶1000 molecules [19]. Several reports have also demonstrated that cancer-specific, methylated DNA can be found in biological fluids, suggesting it could be a useful marker for non-invasive diagnosis [28], [32], [33].

The importance of early detection to improving GC survival outcomes and the promising evidence of DNA methylation as biomarkers is the motivation for this study. Despite growing evidence of the clinical potential of DNA methylation, many inconsistent results can be observed across studies. Hence, this study was undertaken to consolidate information on the clinical potential of methylation in GC by means of a meta-analysis, and to suggest which candidate methylation events deserve further evaluation as clinically relevant biomarkers for the disease.

## Materials and Methods

### Identification and eligibility of studies

A systematic literature search in PubMed for articles published up to October 27, 2011 was performed using ‘“gastric cancer” AND “methylation”’ as the search terms. No restrictions were used during the search in PubMed and the resulting studies were manually curated according to their relevance to GC DNA methylation. These included studies of GC in the areas of hypermethylation and hypomethylation/demethylation of global and target-specific regions. The title and abstract of the papers identified in the initial search were assessed for appropriateness to the aims of this paper. All potentially relevant articles were then evaluated in detail and additional, relevant studies were identified from the citations within these articles. Since the focus of this paper was on human gene methylation, studies that analysed methylation of *H. pylori* and *Epstein-Barr* virus (EBV) genomes in GC progression were not considered.

### Study selection and data annotation

Meta-analyses summarizing frequencies in tumour and normal gastric tissue were restricted to data from case-control studies that reported the frequency of methylation of individual genes in respective groups. Data from reviews and meta-analyses of the same studies were not considered. Information on the first author, year of publication, gene(s) analysed, size of study population, frequency of methylated cases and controls, methods employed for DNA methylation analysis and the sample type were recorded for each study (Table S1, S2). Other relevant details such as the type of lesion, *H. pylori* status, Lauren classification and CpG island methylator phenotype (CIMP) status were also recorded where available. Clinical cases were grouped into eight categories, namely (1) normal mucosa from non-cancer subjects, (2) matching normal mucosa from cases with tumour, (3) chronic gastritis, (4) intestinal metaplasia, (5) dysplastic adenoma, (6) adenocarcinoma, (7) early GC and (8) advanced GC. Due to the small number of studies available, clinical cases were not further classified into *H. pylori* positive/negative cases or intestinal/diffuse type GC and non-cancer subjects were not further classified into matched/unmatched controls. For the same reason, subgroup analysis based on the different stages of precancerous to cancerous lesions was not performed. For consistency, a single gene name based on HUGO nomenclature was assigned to genes with multiple designations.

### Meta-analysis

Meta-analyses were performed using Review Manager 5 (The Cochrane Collaboration, Copenhagen, Denmark). Mantel-Haenszel odds ratios (ORs) were computed for each gene by applying the random-effects model. Homogeneity amongst studies for the same gene was assessed on the basis of the χ^2^ test using the Cochran Q statistic. The I^2^ statistic, which measures the extent of inconsistency between studies, was also assessed. The 95% confidence interval of the odds ratio was used to evaluate differences between groups. If the confidence intervals did not overlap, the two odds ratios were significantly different at the 10% level (since 1−(0.95*0.95)≈0.9). Funnel plots as well as Begg's tests were used to check for publication bias [34]. Publication bias was considered significant when the p-value was <0.1 [35].

## Results

### Study characteristics

**Figure 1:**
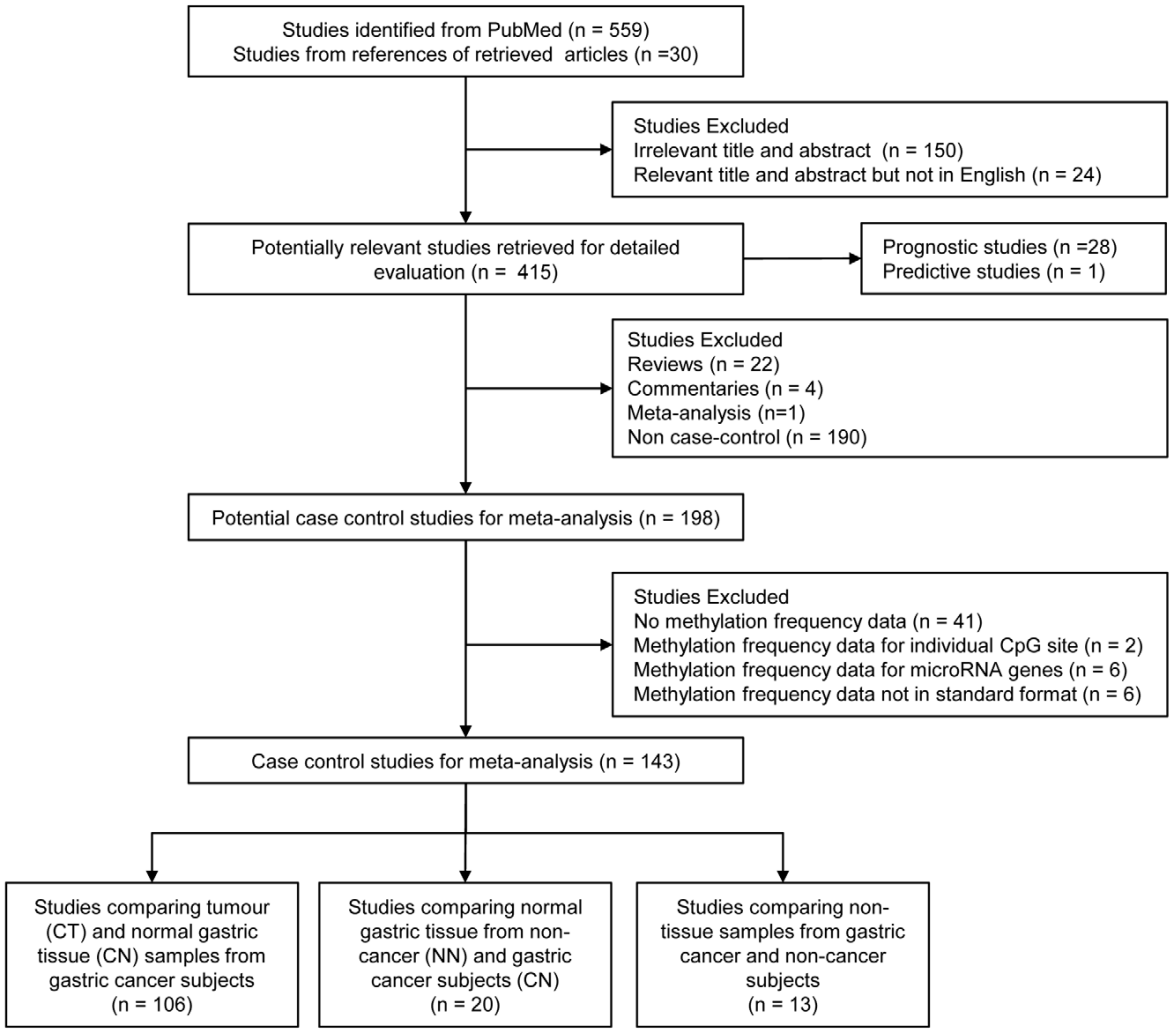
Flow diagram of the literature search strategy and assessment of studies identified for systematic review. Data from some studies was used in multiple meta-analyses, as they reported on more than one case-control analysis considered.

The article selection process used in this study is summarised in Figure 1. A total of 559 studies were identified from PubMed, and an additional 30 studies were further identified from citations of the initial retrieved publications. Based on the appropriateness of the title and abstract to the study objectives, 415 papers were selected for further detailed evaluation. Of these, 190 non case-control studies, 22 reviews, 4 commentaries and 1 meta-analysis were excluded from case-control meta-analyses. Other case-control studies were also excluded, as they were studies of non-protein coding genes (e.g. methylation of micro-RNA genes), the presentation of data was not suitable (e.g. data on individual CpG sites, and demethylation or hypomethylation only) or frequency data was lacking. In total, 143 case-control studies reporting the methylation frequency of 142 individual genes were considered for meta-analyses. The data from some studies was used in more than one meta-analysis, as they contained data on multiple sample type comparisons considered in this study.

### Genes differentially methylated between tumour and normal gastric tissue from gastric cancer subjects

A total of 106 case-control studies reporting on the frequency of methylation in 122 genes in tumour and normal tissue samples from GC subjects were identified for meta-analysis (Table S1). Methylation of 77 of the 122 genes was significantly different between the samples (Table 1), of which data for 62 was derived from a single study only. Methylation was significantly higher in tumour in 70 genes, and in normal tissue in 7 genes.

**Table 1:** Genes differentially methylated in case-control studies of tumour and normal gastric tissue from GC subjects.

Gene	Studies	Overall OR (95% CI)^1^	Gene	Studies	Overall OR (95% CI)^1^	Gene	Studies	Overall OR (95% CI)^1^
*MLH1*	15	3.16 [1.80, 5.56]	*CMTM3*	1	4.80 [1.65, 13.98]	*MT1G*	1	0.06 [0.01, 0.28]
*p16*	13	3.10 [1.22, 7.85]	*CST6*	1	13.44 [4.72, 38.32]	*p15*	1	11.40 [3.09, 42.03]
*CHFR*	10	8.66 [4.51, 16.64]	*CXCL12*	1	14.85 [4.24, 52.03]	*PCDH10*	1	28.67 [8.51, 96.56]
*RUNX3*	9	4.31 [2.14, 8.70]	*CYP1B1*	1	34.55 [1.89, 631.93]	*PDX1*	1	69.41 [3.89, 1239.18]
*APBA1*	3	3.70 [1.28, 10.66]	*DAB2IP*	1	9.75 [2.00, 47.50]	*PRDM5*	1	89.00 [5.29, 1496.15]
*APBA2*	3	8.11 [3.04, 21.63]	*DKK3*	1	3.95 [2.36, 6.60]	*PTCH1a*	1	163.86 [10.02, 2679.17]
*HLTF*	3	7.34 [2.75, 19.58]	*DLC1*	1	83.98 [5.04, 1397.98]	*RAB32*	1	36.89 [2.12, 641.33]
*MGMT*	3	5.18 [1.22, 21.98]	*DLEC1*	1	55.08 [3.02, 1003.70]	*RARB*	1	5.39 [1.69, 17.22]
*MINT31*	3	2.76 [1.04, 7.33]	*FLNC*	1	8.10 [1.78, 36.91]	*RARRES1*	1	0.11 [0.03, 0.41]
*PRDM2*	3	5.19 [2.25, 11.94]	*GRIK2*	1	25.00 [4.81, 129.86]	*RASSF1A*	1	11.50 [4.49, 29.44]
*SFRP1*	3	3.43 [1.55, 7.61]	*HAND1*	1	30.60 [1.79, 522.23]	*RNF180*	1	145.85 [8.70, 2446.27]
*SFRP5*	3	0.50 [0.27, 0.91]	*HIC1*	1	4.75 [1.40, 16.14]	*SOCS1*	1	5.76 [1.59, 20.92]
*ITGA4*	2	25.50 [6.10, 106.61]	*HLA-A*	1	6.22 [2.45, 15.79]	*SPINT2*	1	113.29 [6.27, 2045.31]
*SFRP2*	2	4.87 [1.01, 23.40]	*HLA-B*	1	8.46 [3.01, 23.74]	*SYK*	1	65.30 [3.85, 1108.43]
*TERT*	2	13.41 [1.57, 114.69]	*HLA-C*	1	14.06 [3.82, 51.67]	*TAC1*	1	4.71 [1.67, 13.32]
*ADRA1B*	1	7.20 [1.24, 41.94]	*HOPX*	1	46.38 [18.09, 118.92]	*TBPL1*	1	0.17 [0.05, 0.53]
*APAF1*	1	4.56 [1.52, 13.73]	*HRASLS*	1	34.19 [2.01, 582.90]	*TCF4*	1	36.00 [5.80, 223.54]
*BCL2*	1	29.33 [6.20, 138.78]	*IGF2*	1	0.05 [0.01, 0.46]	*TFPI2*	1	17.50 [3.31, 92.47]
*BDNF*	1	34.55 [1.89, 631.93]	*IQGAP2*	1	22.62 [1.28, 399.63]	*THBD*	1	4.62 [1.27, 16.84]
*BNIP3*	1	143.08[8.54, 2396.38]	*KL*	1	0.18 [0.03, 0.98]	*THBS1*	1	16.30 [3.75, 70.87]
*BTG4*	1	84.14 [4.61, 1534.87]	*KLF4*	1	33.00 [1.06, 1023.56]	*TIMP3*	1	9.00 [1.46, 55.48]
*CACNA1G*	1	22.15 [2.58, 189.95]	*LMX1A*	1	12.97 [4.97, 33.83]	*TSPYL5*	1	10.97 [3.43, 35.13]
*CACNA2D3*	1	7.71 [2.19, 27.12]	*LOX*	1	5.17 [1.42, 18.79]	*UCHL1*	1	8.75 [2.19, 34.90]
*CADM1*	1	36.64 [2.16, 621.68]	*LRP1B*	1	5.20 [2.55, 10.64]	*XRCC1*	1	12.54 [3.98, 39.53]
*CDH5*	1	79.22 [3.87, 1622.84]	*MINT12*	1	3.42 [1.30, 9.00]	*ZIC1*	1	1065.00 [49.40, 22961.75]
*CDKN1C*	1	0.02 [0.00, 0.20]	*MINT25*	1	204.60 [12.28, 3409.55]			

1 Odds ratio (OR) describes the likelihood of gene methylation observed in tumour compared to normal gastric tissue. Only the genes for which there was a significant difference in methylation frequency between the two groups are displayed (p<0.05). Genes for which there was no significant difference are listed in Table S1.

### Genes differentially methylated in normal gastric tissue from GC and non-cancer subjects

Twenty case-control studies comparing the frequency of methylation in 34 genes between normal tissue samples from GC and non-cancer subjects were identified for meta-analysis (Table S2). Of these, methylation in 15 genes was significantly different, of which the data from 11 genes were derived from a single study only (Table 2). For all 15 genes, methylation was higher in normal tissue from GC compared to non-cancer subjects. Considering methylation events examined in more than one study, 4 (*p16*, *CDH1*, *DAPK*, *CHFR*) were found to be significantly different.

**Table 2:** Genes differentially methylated in case-control studies of normal tissue, serum and plasma from gastric cancer and non-cancer subjects.

Gene	Studies	Overall OR (95% CI)^1^
**Normal gastric tissue**		
*p16*	6	2.91 [1.35, 6.30]
*CDH1*	3	8.54 [5.17, 14.09]
*DAPK*	3	6.42 [3.89, 10.60]
*CHFR*	2	8.55 [1.49, 49.13]
*BX161496*	1	9.35 [2.50, 35.04]
*CDH4*	1	81.67 [3.98, 1673.88]
*CYP26B1*	1	77.50 [8.55, 702.90]
*GRIN2B*	1	12.29 [1.43, 105.45]
*KCNA4*	1	41.08 [8.19, 205.99]
*RELN*	1	25.00 [1.03, 608.09]
*RUNX3*	1	23.10 [1.35, 396.36]
*SFRP1*	1	97.36 [4.93, 1922.50]
*SFRP5*	1	21.00 [1.13, 390.57]
*TMEFF2*	1	12.86 [1.52, 108.54]
*WT1*	1	11.43 [3.09, 42.27]
**Serum**		
*CDH1*	3	15.27 [2.77, 84.28]
*p16*	5	12.69 [3.49, 46.10]
*DAPK*	1	56.72 [3.30, 974.73]
*SULF1*	1	5.19 [1.28, 21.08]
*p15*	1	75.94 [4.42, 1305.71]
*SFRP2*	1	71.15 [3.67, 1379.46]
*SOCS1*	1	25.00 [1.20, 520.73]
**Plasma**		
*MGMT*	1	4.08 [1.12, 14.86]
*p15*	1	4.50 [1.12, 18.13]
*RNF180*	1	164.59 [9.37, 2891.77]
*RPRM*	1	191.33 [30.01, 1220.01]

1 Odds ratio (OR) describes the likelihood of gene methylation observed in samples from gastric cancer compared to non-cancer subjects. Only genes in which there were significant differences in methylation between the two groups are displayed (*p*<0.05). Genes for which there was no significant difference are displayed in Table S2.

### Genes differentially methylated in non-tissue samples from GC and non-cancer subjects

A total of 26 studies reporting on methylation in 29 genes in clinical samples other than gastric tissue, including whole blood, plasma, serum, gastric washes, peritoneal fluid and faecal samples from GC subjects were identified. Of these, 13 studies examining methylation of a total of 14 genes in either serum, faecal or plasma samples were of a case-control design and hence suitable for meta-analysis (Table S2). Significantly different methylation frequencies were observed in 4 genes in plasma samples, 7 genes in serum samples and 0 in faeces (Table 2). *p15* was a common gene identified in studies of plasma and serum, making it 10 unique genes altogether significantly different in methylation frequency in blood samples. Methylation in only two genes (*CDH1, p16*) was examined in more than one study, and both were significantly different in frequency in the samples from GC and non-cancer subjects in meta-analysis.

### Methylation as a prognostic and predictive marker for GC

A total of 28 studies were identified that investigated gene methylation in 40 genes in relation to the survival outcome of GC subjects (Table 3). Of the 40 genes studied, only 5 were examined in multiple studies. Meta-analyses could not be performed on this series of studies due to the irregular reporting of hazard ratios. A significant association with survival was reported for methylation in 18 of the 40 (45%) genes, although inconsistency in the finding of significant differences was observed for all gene examined in multiple studies.

**Table 3:** Summary of gene methylation and GC prognosis in the component studies.

Gene	Studies	Author Year	Cases	Overall Survival
*APBA1*	1	An 2005	82	NS
*APBA2*	1	An 2005	81	NS
*APC*	1	Leung 2005	58	Poor OS with CDH1 (p = 0.006)
*BNIP3*	1	Sugita 2011	80	Poor (p = 0.031)
*BCL6B*	1	Xu 2011	309	Poor (p = 0.025 (cohort I)/p = 0.016 (cohort II))
*CACNA2D1*	1	Wanajo 2008	53	Poor (p = 0.003)
*CACNA2D3*	1	Wanajo 2008	53	NS
*CDH1*	6	Graziano 2004	73	Poor (p<0.001)
		Ikoma 2006	97	Poor (p<0.05)
		Leung 2005	58	Poor OS with APC (p = 0.006)
		Napieralski 2007	61	NS
		Tahara 2010	126	NS
		Zazula 2006	84	NS
*CST6*	1	Chen 2010	52	Poor (p = 0.020)
*DAPK*	4	Chan 2005	102	Poor^1^ (p = 0.0141)
		Kato 2008	81	Poor OS with TMS1 (p = 0.0003)
		Tahara 2010	126	Poor (p = 0.017)
		Sugita 2011	80	NS
*DKK3*	1	Yu 2009	104	Poor (p<0.0001)
*DMRT1*	1	Jee 2009	152	NS
*EBF3*	1	Kim 2011	104	Poor (p = 0.038)
*FBP1*	1	Liu 2010	46	Poor (p = 0.01)
*GPX1*	1	Jee 2009	152	NS
*GPX3*	1	Jee 2009	152	NS
*HOPX*	1	Ooki 2010	90	Poor (p = 0.029)
*IGFBP6*	1	Jee 2009	152	NS
*IQGAP2*	1	Jin 2008	36	Poor (p = 0.0029)
*IRF7*	1	Jee 2010	152	NS
*KL*	1	Wang 2011	99	Poor (p = 0.025)
*LOX*	1	Napieralski 2007	61	NS
*MAGEA1/A3*	1	Honda 2004	84	NS
*MAL*	1	Buffart 2008	179	Better (p = 0.03)
*MGMT*	2	Napieralski 2007	61	NS
		Park 2001	79	Poor (p<0.02)
*MINT25*	1	An 2005	82	NS
*MINT31*	1	An 2005	82	Better (p = 0.04)
*MLH1*	3	An 2005	82	NS
		Ishiguro 2003	102	Better (p<0.05)
		Leung 2005	58	NS
*p14*	1	Tahara 2010	126	NS
*p16*	4	An 2005	82	NS
		Ikoma 2006	97	NS
		Napieralski 2007	61	NS
		Tahara 2010	126	NS
*PCDH10*	1	Yu 2009	31	NS
*PTGS2*	1	de Maat 2007	40	Better (p = 0.03)
		de Maat 2007	137	Better (p = 0.01)
*RARB*	1	Ikoma 2006	97	NS
*SLC19A3*	1	Liu 2009	101	NS
*TFPI2*	1	Jee 2009	152	Poor (p = 0.023)
*TIMP3*	1	Leung 2005	58	NS
*TMEFF2*	1	Napieralski 2007	61	NS
*TMS1*	1	Kato 2008	81	Poor OS with DAPK (p = 0.0003)
*YWHAQ*	1	Napieralski 2007	61	NS

1 The prognostic outcome was based on disease-free survival (DFS). The brackets displayed the p-value for studies that showed significance. NS: Not significant.

Five studies reported on associations between the survival in GC subjects receiving a specific chemotherapy treatment and methylation of 10 genes including *TMS1*, *DAPK*, *LOX*, *MGMT* and *CHFR* [36]–[40]. In all 5 studies, subjects with methylation had a worse survival than those without methylation. One study examined survival differences between subjects treated with and without chemotherapy according to *p16* methylation status [41]. In this study, subjects without *p16* methylation that received chemotherapy had a better survival than those that did not, whereas for subjects with *p16* methylation, there was no significant difference according to treatment status.

### Effect of analytical variability and publication bias

During the annotation of studies, considerable heterogeneity was observed in many study parameters, including the assays used, CpG sites interrogated, stages of disease examined, and the sample types used (e.g. fresh, frozen or paraffin-embedded tissue). Twelve different assays were used for the evaluation of methylation, with methylation-specific PCR (MSP) being the most common. To examine the influence of methylation assay on study results, data from methylation-specific PCR and quantitative methylation-specific PCR (e.g. Methylight) analysis were compared for the gene most frequently examined in studies comparing methylation between gastric tumour and normal tissue (*MLH1*, 13 case-control studies). There was no statistical difference (*P*<0.05) in the 95% confidence intervals of the methylation frequencies reported using the two assays (Figure S1). No significant differences according to sample type (frozen vs. paraffin-embedded tissue), stage of disease, or CpG sites interrogated were also observed in these studies (results not shown).

To test for publication bias, data from the same 13 studies on *MLH1* methylation mentioned above was also examined. A trend towards positive reporting was observed in funnel plot (Figure S2) and Begg's test analysis. However, these results should be interpreted with caution as most studies were of small sample size, and a minimum of 20 studies is usually recommended for a reliable analysis of publication bias [42].

## Discussion

Numerous studies have implicated aberrant DNA methylation at numerous genes in different samples and models of gastric tumourigenesis [1], [5]–[10], [12]–[14], [16], [18]–[22], [24], [43]. This implication has in turn given rise to the notion that methylation could be a useful biomarker for improving the clinical management of GC [11], [15], [32], [44], [45]. To date however, this potential has not been realized, presumably due to a lack of relevant evidence to support the testing of methylation in the clinic.

In this study, a comprehensive review of all publications on the frequencies and associations of methylation in gastric cancer clinical samples was performed to consolidate information in the field. Meta-analyses were conducted where possible to gain an objective consensus from repeatedly investigated events. From the analysis, lists were generated of genes significantly differentially methylated between tumour and normal tissue sample from GC subjects (Table 1), and normal tissue and/or blood from GC and non-cancer subjects (Table 2), each with methylation events annotated for their strength of association and frequency of analysis. Findings from studies on the prognostic and predictive significance of methylation events were also reviewed (Table 3). These lists and additional supplementary data (Tables S1, S2) should provide useful information from which to better assess the clinical potential of the respective events, and prioritize further work.

Comprising 77% (101/132) of case-control analyses (Figure 1), the largest group of studies reviewed were those comparing the frequency of methylation in tumour and normal gastric tissue from GC subjects. The review identified 77 significant gene methylation events, confirming by meta-analysis at the same time the significantly different methylation of a number of genes commonly implicated in tumourigenesis, including *MLH1*, *p16*, and *CHFR* and *RUNX3* (Table 1). These events represent useful tools for better understanding gastric tumourigenesis and potentially identifying new therapeutic strategies [3], [28]. From the perspective of risk markers however, these events can only be considered a first pool of candidates to test further in more clinically relevant analyses, as the events on their own only identify gastric tumour samples that are already histologically diagnosable.

From studies comparing methylation levels in normal tissue, plasma and serum from GC and non-cancer subjects, 15, 4 and 7 (Table 2) significantly different gene methylation events respectively were identified. Genes with both established roles (such as *p16*, *CDH1*, *DAPK*, *RUNX3*, *p15*) and lesser-known roles (such as *BX161496*, *SULF1*, *RPRM*) in tumourigenesis were identified. A number of events were in common between studies on normal tissue and blood, such as methylation at *p16*, *CDH1*, *DAPK*. These events are clinically promising, as they demonstrate discriminative capabilities for estimating GC risk from samples that can be obtained in current routine practice, such as during endoscopic screening (tissue), or population or clinical screening (blood).

The hypothesis that gene silencing by methylation may also determine severity of disease [27] has also prompted numerous investigations of gene methylation associations with survival in GC. In this review, 28 studies reporting on the association of survival of GC subjects and methylation at 40 genes were also identified. In support of the hypothesis, numerous significant associations between methylation and poor survival were recorded, primarily at tumour suppressor genes (Table 3). Associations between methylation and better survival were also reported for four genes (*PTGS2*, *MINT31*, *MLH1*, *MAL*), presumably reflecting that suppression of oncogenic activity by gene methylation can also occur. However, a considerable lack of independent study and replication of associations for most of the genes studied (Table 3) highlights a need for further investigation in this aspect of methylation in GC.

Data from five studies on patients receiving chemotherapy also has suggested that DNA methylation at *CHFR*, *DAPK*, *TMS1* could be useful predictors of response to chemotherapy [36]–[40]. However, from the design of these analyses, it is difficult to determine whether the survival differences were due to inherent prognostic differences or were a function of a treatment interaction, or both. In a study of a different design, Mitsuno *et al.* reported that patients with p16 methylation gained a survival benefit from chemotherapy, while those without methylation did not [41]. This result suggests that p16 methylation may be a useful marker for predicting response to chemotherapy, and provides evidence of a treatment interaction. However, this study only examined 56 subjects in a retrospective analysis, and much further work is required to confirm these findings.

The findings of this study highlight a promising potential for DNA methylation in GC risk prediction, prognostication and prediction of treatment response. However, many issues relevant to clinical implementation remain unaddressed by the studies. Methodologically, the studies inadequately define optimal approaches for analysis, due to their large variability in assays, PCR primers and probes, PCR conditions, and thresholds for positivity used. Most studies (102/143, 71%) have been based on methylation-specific PCR, for which the non-quantitative nature of analysis presents difficulties to quality control and standardization. With methylation a dynamic event, protocols for sampling are also in need of clarification, both with respect to the region or site of sampling, and time of sampling. The distance of normal tissue from tumour [46], and time of sampling [21], [22] have all been documented to significantly influence methylation levels.

The large variability in the genes and gene panels examined between studies, combined with a lack of validation in independent series and characterization of test performance characteristics, also makes it difficult to define a clinically relevant test. Variation in the interrogation of often functionally different CpG sites [47]–[49] between studies of the same gene provides additional complications. Moreover, all the gene methylation events examined in multiple studies and significant associated with GC (including *p16*, *DAPK*, *CHFR*, *MLH1*, *RUNX3*) have been implicated as risk markers of many other cancer types [26], [27], [50], raising questions about the interpretation of their detection in asymptomatic individuals.

Of further consideration are the co-variates of analysis that would be analyzed with methylation. Methylation has been associated with many demographic, clinical and molecular features, including age, gender, smoking, intestinal metaplasia, host genetics, and *H. pylori* and Epstein Barr virus status [1], [24]. In addition to direct associations, many studies have also reported modifying interactions between many of these features and methylation on GC risk [51]. Methylation events themselves also may be linked and interact with each other [50], presenting a challenge to define an optimal panel of methylation markers as well. A CpG island methylator phenotype (CIMP), consisting of distinct subtypes of GC with co-ordinated methylation patterns, has been described [52]–[55], although the evidence for CIMP in GC is not as convincing as for colorectal cancer [56], [57].

In conclusion, the results of this study summarize a promising value for DNA methylation to the risk prediction, prognostication and prediction of response to chemotherapy of GC. However, significant methodological and validation issues remain to be addressed to provide the data that will enable this information to be considered for the clinic. This includes the analysis of larger independent sample series, application of standardized methods, adjustment for co-variates in multivariate analysis, greater definition of outcome endpoints and adjustment for the effect of treatment intervention. The realization of the potential of DNA methylation to GC clinical management awaits their resolution.

## Supporting Information

Figure S1 **Forest-plot of methylated studies comparing** ***MLH1*** **methylation between tumour and normal tissue from GC subjects according to use of methylation-specific PCR and quantitative methylation-specific PCR.**
(TIF)

Figure S2 **Funnel plot of all 13 studies of** ***MLH1*** **methylation in tumour and normal gastric tissue from GC subjects to evaluate publication bias.** The vertical line indicates the pooled estimate of the overall OR and the sloping lines represent the 95% confidence interval.
(TIF)

Table S1 **List of methylated genes and their component studies for comparing differences between tumour and normal gastric tissue from gastric cancer subjects.** Meta-analysis odd ratios (OR) and 95% confidence interval were computed for all methylated genes analysed. Red and bold fonts were used to indicate significant differences between groups. BS-SSCP: bisulfite single-stranded conformation polymorphism; Bseq: bisulfite sequencing; COBRA: combined bisulfite restriction analysis; DPHLC: Denaturing High Performance Liquid Chromatography; FFPE: formalin-fixed paraffin embedded; HRM: high resolution melting; MSP: methylation-specific polymerase chain reaction; PCR: polymerase chain reaction; QMSP: quantitative methylation-specific polymerase chain reaction.
(XLS)

Table S2 **List of methylated genes and their component studies for comparing differences in normal tissue, plasma and serum between gastric cancer and non-cancer subjects.** Meta-analysis odd ratios (OR) and 95% confidence interval were computed for all methylated genes analysed. Red and bold fonts were used to indicate significant differences between groups. COBRA: combined bisulfite restriction analysis; MSP: methylation-specific polymerase chain reaction; QMSP: quantitative methylation-specific polymerase chain reaction.
(XLS)
