# Supplementary figures and images for: Clinical Potential of DNA Methylation in Gastric Cancer: A Meta-Analysis
Source: PLoS One. 2012 Apr 27;7(4):e36275. doi: 10.1371/journal.pone.0036275 (PMC3338684; doi:10.1371/journal.pone.0036275)

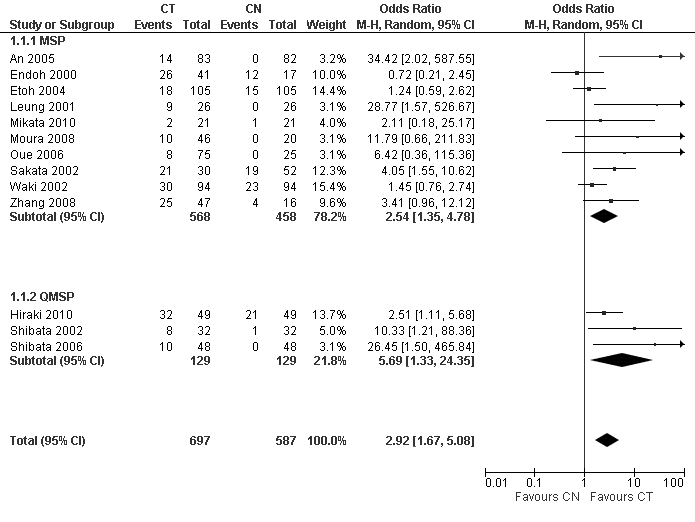

Supplement: Figure S1 — Forest-plot of methylated studies comparing MLH1 methylation between tumour and normal tissue from GC subjects according to use of methylation-specific PCR and quantitative methylation-specific PCR. (TIF) [file pone.0036275.s001.tif]

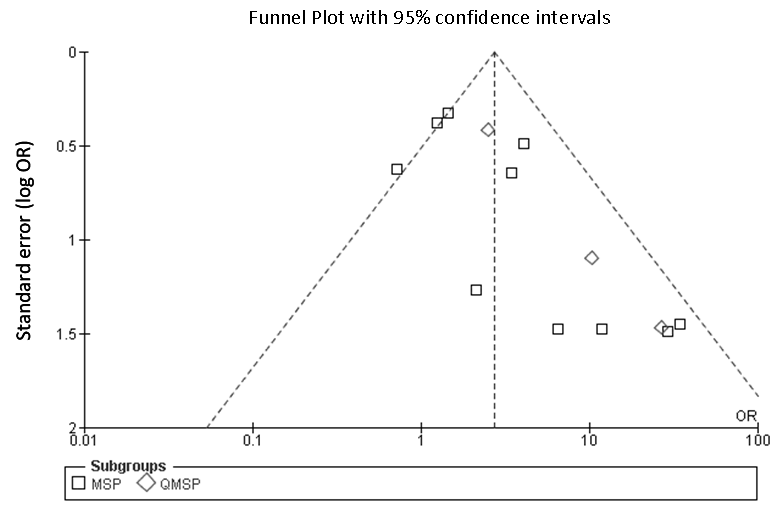

Supplement: Figure S2 — Funnel plot of all 13 studies of MLH1 methylation in tumour and normal gastric tissue from GC subjects to evaluate publication bias. The vertical line indicates the pooled estimate of the overall OR and the sloping lines represent the 95% confidence interval. (TIF) [file pone.0036275.s002.tif]
